# Single-Cell Transcriptomics and In Vitro Lineage Tracing Reveals Differential Susceptibility of Human iPSC-Derived Midbrain Dopaminergic Neurons in a Cellular Model of Parkinson’s Disease

**DOI:** 10.3390/cells12242860

**Published:** 2023-12-18

**Authors:** Lucia F. Cardo, Jimena Monzón-Sandoval, Zongze Li, Caleb Webber, Meng Li

**Affiliations:** 1 Dementia Research Institute, School of Medicine, Cardiff University, Hadyn Ellis Building, Maindy Road, Cardiff CF24 4HQ, UK; cardolf@cardiff.ac.uk (L.F.C.); monzonsandovalj@cardiff.ac.uk (J.M.-S.); liz55@cardiff.ac.uk (Z.L.); 2 Neuroscience and Mental Health Innovation Institute, School of Medicine, Cardiff University, Hadyn Ellis Building, Maindy Road, Cardiff CF24 4HQ, UK

**Keywords:** CRISPR/Cas9, midbrain dopaminergic neuron, genome editing, human pluripotent stem cell, in vitro differentiation, Parkinson’s disease, single-cell RNA sequencing

## Abstract

Advances in stem cell technologies open up new avenues for modelling development and diseases. The success of these pursuits, however, relies on the use of cells most relevant to those targeted by the disease of interest, for example, midbrain dopaminergic neurons for Parkinson’s disease. In the present study, we report the generation of a human induced pluripotent stem cell (iPSC) line capable of purifying and tracing nascent midbrain dopaminergic progenitors and their differentiated progeny via the expression of a Blue Fluorescent Protein (BFP). This was achieved by CRISPR/Cas9-assisted knock-in of BFP and Cre into the safe harbour locus AAVS1 and an early midbrain dopaminergic lineage marker gene LMX1A, respectively. Immunocytochemical analysis and single-cell RNA sequencing of iPSC-derived neural cultures confirm developmental recapitulation of the human fetal midbrain and high-quality midbrain cells. By modelling Parkinson’s disease-related drug toxicity using 1-Methyl-4-phenylpyridinium (MPP^+^), we showed a preferential reduction of BFP^+^ cells, a finding demonstrated independently by cell death assays and single-cell transcriptomic analysis of MPP^+^ treated neural cultures. Together, these results highlight the importance of disease-relevant cell types in stem cell modelling.

## 1. Introduction

Parkinson’s disease (PD) is the second most common neurodegenerative disorder, affecting 2–3% of the population over 65 years of age and with an incidence increase of 5–10 fold in the later decades of life [1]. The main pathological features of PD are the loss of midbrain dopaminergic (mDA) neurons in the substantia nigra pars compacta (SNpc), which project to the striatum and cortex. Current treatment for PD is mainly pharmacological intervention to counterbalance the dwindling supply of striatal dopamine and in some cases managed by a type of surgery called deep brain stimulation. However, all currently available treatments are palliative, and there are no means to stop disease progression, cure, or prevent PD.

Human pluripotent stem cells (PSCs), including embryonic stem cells and induced pluripotent stem cells, can generate an unlimited amount of authentic mDA neurons during in vitro differentiation. These, in vitro, produced mDA cells are amenable to pharmacological and/or gene expression perturbations to mimic PD-related cellular pathologies and thus present a valuable model for elucidating the molecular mechanisms underlying PD and advancing therapeutic development. MDA neural progenitors can now be induced at a high efficiency from hPSCs and clinical trials using such preparations have been approved for PD cell therapy [2,3,4,5,6,7,8,9]. However, neuronal derivatives expressing the cardinal mDA markers (e.g., PITX3) are often present in much lower proportion relative to the number of progenitors at early stages, either after differentiation in vitro or following transplantation in rodent brains. For studies aiming to evaluate the effects of drugs or PD pathogenic genetics on mDA neurons and/or late mDA precursors using RNA sequencing approaches, the low neuronal content incurs significant unwanted cellular heterogeneity. Therefore, PSC lines with a genetically engineered cell type-specific fluorescent reporter would facilitate the isolation of desired cell identity using a fluorescent-activated cell sorter (FACS). For example, a tyrosine hydroxylase (TH)-based reporter system has been employed for purifying dopamine neurons from mixed PSC-derived neuronal cultures and for studying the migration and integration of transplanted cells in the host brains [10,11,12] or isolating TH-expressing cells after fixation and immunostaining with fluorophore-conjugated antibodies [13,14]. However, TH expression is not limited to SNpc (A9) but is also expressed in other catecholaminergic neurons [15], while its late expression in postmitotic cells does not meet the requirement for isolating mDA progenitors and for genetic or pharmacological interrogation prior to or during their differentiation into postmitotic mDA neurons. Moreover, there are currently no available hPSC tools designed for genetic tracking of derivatives of mDA progenitors for in-depth investigations into their developmental fate and characteristics other than the immediate neuronal derivatives of mDA.

During development, the specification of mDA fate in ventral midbrain progenitors is marked by the expression of transcription factor LMX1A [16,17,18,19]. Using CRISPR/Cas9 assisted genome editing technology, we knocked in a Cre and silent blue fluorescent protein (BFP) expression unit into the LMX1A and AAVS1 locus, respectively, in the KOLF2-C1 human iPSC line (HPSI0114i-kolf_2, https://hpscreg.eu/cell-line/WTSIi018-B (accessed on 9 November 2023), reference [20]). We show here that LMX1A^+^ mDA neural progenitors derived from the tracer lines can be purified based on BFP expression via FACS with BFP expression maintained in their differentiated progeny regardless of their own LMX1A expression status. Single-cell RNA sequencing (scRNAseq) analysis confirmed the authenticity of iPSC mDA identity, and preferential vulnerability of BFP^+^ neurons to toxicity of 1-Methyl-4-phenylpyridinium (MPP^+^).

## 2. Materials and Methods

### 2.1. PSC Culture and mDA Differentiation

KOLF2-C1 and genome-edited KOLF2-C1 derivatives were maintained on Matrigel-coated plates in Essential 8 media (TeSR-E8, Stemcell Technologies, Vancouver, BC, Canada). All iPSCs were passaged via manual dissociation using Gentle Cell Dissociation Reagent as described previously [21]. IPSC differentiation toward mDA fate follows a protocol combining key features of Kriks et al. and Jagger et al. [2,3]. Briefly, iPSCs were pre-plated on growth factor-reduced matrigel in TeSR-E8. Neural differentiation was initiated when cells reached >80% confluence by switching to DMEM-F12/Neurobasal (2:1) supplemented with N2 and B27 (N2B27). For the first 7 days, cultures were supplemented with SB431542 (10 µM, Tocris, Bristol, UK), LDN-193189 (100 nM, StemGene, Manchester, UK), SHH-C24II (200 ng/mL, BioTechne, Minneapolis, MN, USA) and Purmorphamine (1 µM, VWR, Radnor, PA, USA). LDN was kept until day 11. On day 3 CHIR99021 (3 µM, Cambridge Bioscience, Cambridge, UK) was added to the media until day 13. Cultures were treated with PD0325901 from day 5 to 9 (1 µM, Cambridge Bioscience, Cambridge, UK). FGF8a was added from day 11 to day 20 (100 ng/mL, Peprotech, Cambridge, UK). Cultures were dissociated at around day 16/17 to single cells using Accutase (Thermo Fisher Scientific, Cambridge, UK) and replated onto poly-D-lysine/laminin-coated plates at a density of 250,000 cells/cm^2^. B27 without vitamin A (Thermo Fisher Scientific) was used for the first 25 days, followed by B27 (Thermo Fisher Scientific) plus BDNF (10 ng/mL, Peprotech), GDNF (10 ng/mL, Cell Guidance System, Cambridge, UK), Ascorbic Acid (200 µM, Sigma-Aldrich, St. Louis, MO, USA), and DAPT (10 µM, Tocris) from day 21 onwards.

Enhanced growth factor containing media was used for FACS purification at day 18 and post-sort culture of sorted BFP^+^, sorted BFP^−^ and unsorted sister control cells. DMEM-F12/Neurobasal basal media was supplemented with normal B27 together with TGFβ3 (1 ng/mL, Peprotech), dbcAMP (500 µM, Sigma-Aldrich), Ascorbic Acid (200 µM), DAPT (10 µM) and GDNF and BDNF at 20 ng/mL each. The concentration of GDNF and BDNF was reduced to 10 ng/mL one week after sorting.

### 2.2. CRISPR/Cas9 Genome Editing of Human iPSCs

**Figure 1:**
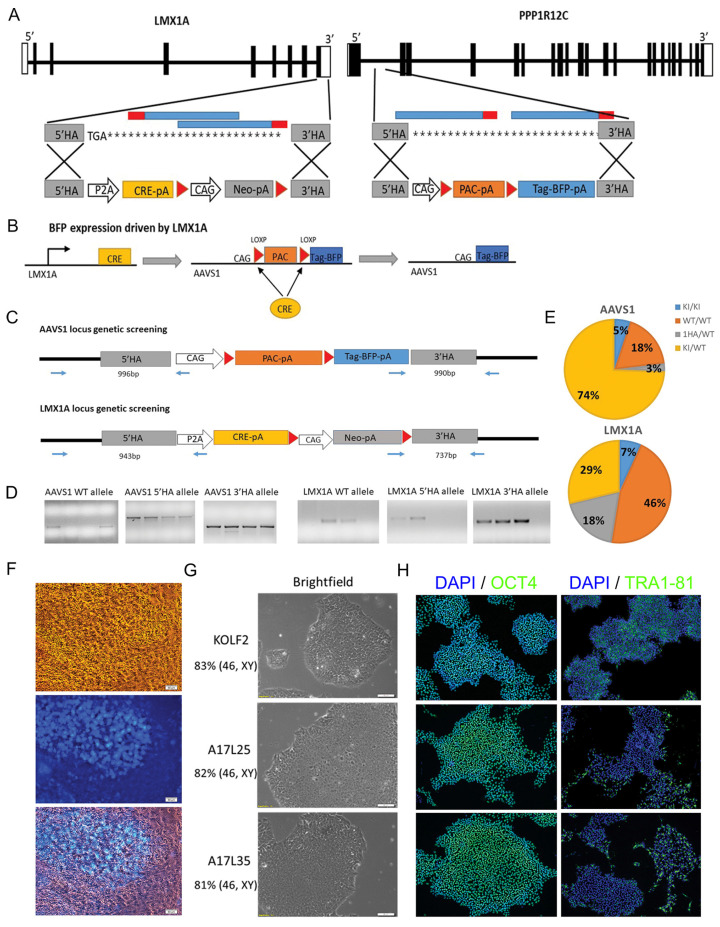
Generation of LMX1A-Cre/AAVS1-BFP tracing lines. (**A**) Schematic illustration of the wild type LMX1A and PPP1R12C (AAVS1) loci and targeting strategy. Exons are indicated as black rectangles. left: LMX1A targeting, the two gRNAs targeting the 3′UTR of LMX1A are indicated in blue with PAM region in red. The P2A-Cre-pA and Neo-pA expression cassettes are flanked by the homologous arms (HA) corresponding to exon 8 immediately upstream of the stop codon and 3′UTR (grey), respectively. Right: AAVS1 targeting, the two gRNAs targeting intron 1 of AAVS1 were indicated in blue with PAM region in red. The homologous arms (HA), indicated in grey, flank a PAC-pA selection cassette and BFP-pA fluorescent tag gene. (**B**) Mechanism of BFP activation driven by LMX1A expression. (**C**) Genomic PCR screening strategy for detecting the targeted clones at the AAVS1 and LMX1A locus, respectively. (**D**) Examples of agarose gels showing amplicons for the WT and HR allele. (**E**) Targeting efficiency for AAVS1 and LMX1A locus, respectively. (**F**) Bright field and fluorescent images of day 10 mDA differentiation cultures showing BFP expression in live cells. Scale bar 50 μm. (**G**) Representative bright field images of iPSC colonies and (**H**) immunostaining of pluripotency markers OCT4 and TRA1-81 with DAPI counterstain for the parental KOLF2 and A17L25 and A17L35 LMX1ACre-BFP lines. Scale bar 50 μm. Karyotyping results are indicated on the left below the corresponding iPSC lines.

Two rounds of CRISPR/Cas9 genome editing were carried out to generate the LMX1A-Cre/AAVS1-BFP tracer lines. The first round of editing concerns the targeted insertion of a BFP expression cassette downstream of loxP flanked puromycin expression unit in the AAVS1 safe harbor locus. In the second round genome editing, a CRE protein expression cassette was knocked in into the LMX1A locus. This strategy allows LMX1A-dependent expression of Cre, which then removes the floxed puromycin cassette sandwiched between the CAG promoter and BFP-poly A sequence in the AAVS1 locus, leading to LMX1A controlled expression of BFP (Figure 1). Detailed description of CRISPR/Cas9 editing, associated genotyping, and quality control analysis is provided in Supplemental Methods and Supplemental Tables S1 and S2.

### 2.3. Immunofluorescence

Cultures were fixed with 4% (*w*/*v*) paraformaldehyde and permeabilized with 0.1% (*v*/*v*) Triton X-100 in PBS (PBS-T). Following blocking with 1% (*w*/*v*) bovine serum albumin and 3% (*v*/*v*) donkey serum, cells were incubated with primary antibodies overnight at 4 °C. After three washes with PBS-T, cells were incubated with complementary Alexa Fluor-conjugated antibodies (1:1000, Life technologies, Carlsbad, CA, USA) for 1 h at room temperature, and then counterstained with DAPI. All antibodies were diluted in PBS-T containing 1% (*w*/*v*) BSA and 1% (*v*/*v*) donkey serum. The primary antibodies used in the study are detailed in Supplemental Table S3.

Images were acquired using a Leica DMI600b inverted microscope. Cell counting was carried out using the CellProfiler 4.2.1 [22] or FIJI 2.9.0 [23] software to analyze 5–6 randomly placed fields of view per replicate. Data were collected from 3 independent differentiation runs for 2 clonal lines each, each with 2 technical replicates. The total sample size (n) is indicated in Figure legends.

### 2.4. Flow Cytometry and FACS Purification

For flow cytometry, cultured cells were dissociated with Accutase as described above and washed twice with DPBS by centrifugation at 900 rpm for 5 min. Cells were resuspended in DPBS and passed through cell strainers with 35 µm mesh (Corning) before being analyzed on a BD LSRFortessa^TM^ cell analyzer. Data were analyzed in FlowJo (BD Biosciences, Wokingham, UK).

For FACS purification, day 18 cultures were processed as described above but resuspended into a sorting buffer containing N2B27, 100 ng/mL FGF8, and ROCK inhibitor Y-27,632 (10 µM, Tocris, Bristol, UK). Sorting was performed on a BD FACSAria III using a 100 M nozzle. Background autofluorescence was compensated for using the KOLF2 parental cell line at the same stage of differentiation, this population was defined as the BFP^−^ gating. BFP^+^ and BFP^−^ cells were isolated using a highly restrictive gating strategy to exclude doublets and debris based on forward- and side-scatter parameters. FACS-isolated cell fractions were replated at 250,000 cells/cm^2^ for neuronal differentiation.

### 2.5. Single Cell RNA Sequencing (scRNAseq) and Associated Data Analysis

Cells were dispensed at a concentration of 30,000 cells/mL onto nanowells using the ICELL8 Single-Cell System (Takara). The cDNA libraries were generated using the SMART-Seq ICELL8 cx Application Kit protocol provided by the manufacturer. Sequencing was carried out on Illumina HiSeq 4000 using 75 bp paired-end sequencing with dual indexing (time course experiment) or the Illumina NovaSeq 6000 platform using 100 bp paired-end sequencing with dual indexing (MPP^+^ experiment). Raw sequencing data were processed using the Mappa pipeline v1.0 (Takara) and aligned to Homo sapiens GRCh38.99 primary assembly with the BFP reporter gene attached to the end of the genome. Counts including introns from the Mappa (1.0) pipeline to infer intron and exon counts as a proxy of un-spliced and spliced RNAs for RNA velocity analysis using velociraptor R package (v 1.10.0). Downstream analysis was performed using the Seurat R package (V3). Detailed methods were described in the supplemental methods.

### 2.6. LDH-Glo™ Cytotoxicity and Annexin V/Dead Cell Apoptosis Assays

Neuronal cultures were treated with 1, 2.5, or 5 mM MPP^+^ (D048, Sigma-Aldrich; prepared fresh following the manufacturer’s instructions) or sterile water as vehicle. A total 24 h after treatment, supernatants were diluted 1:10 in storage buffer. LDH detection reagent and quantification were performed following the manufacturer’s instruction (J2380, Promega, Hampshire, UK). The essay was carried out using 3 technical replicates per culture well. Luminescence was recorded after 45 min incubation at room temperature using a CLARIOstar plate reader (BMG Labtech, Aylesbury, UK) with the following optic settings: emission wavelength 580 nm, emission bandwidth 80 nm, gain 3600, and focal height 10 mm.

Annexin V dead cell assay was carried out using the Alexa fluor 488 Annexin V/Dead cell apoptosis kit (V13245, Invitrogen, Waltham, MA, USA). Working reagents were prepared following the manufacturer’s instructions. Cultures were washed with PBS and dissociated into single cells using Accutase for 10 min at 37 °C followed by gently pipetting and another 2 min of incubation at 37 °C. Accutase reaction was stopped using the same volume of media and the cells were washed by centrifugation in PBS and re-suspended in 1X annexin-binding buffer. The cell suspension was then incubated with 5% (*v*/*v*) Alexa Fluor^®^ 488 Annexin V and 10 μg/mL PI at room temperature for 15 min. After this incubation period, 4 times the volume of 1X annexin-binding buffer was added. The stained cells were then processed using the BD LSRFortessa cell analyzer by measuring the fluorescence emission at 530 nm and 575 nm with the gating strategy shown in Figure S2 and data analyzed in FlowJo. The assays were performed in three independent cultures.

### 2.7. Statistical Analysis

Statistical analyses were performed using IBM SPSS 23 software. Student’s *t*-test or Mann–Whitney U test was used for comparisons between the two groups. One-way ANOVA with Bonferroni Post Hoc or Kruskal–Wallis Test was used for comparisons between the three groups. Statistically significant differences were considered when *p*-value ≤ 0.05.

## 3. Results

### 3.1. Generation of Human iPSC LMX1A-Cre/AAVS1-BFP Lineage Tracer Line

The LMX1A-Cre/AAVS1-BFP tracer line was generated following two rounds of CRISPR/Cas9 genome editing in the KOLF2-C1 human iPSC line (to be referred to as KOLF2 subsequently): (1) knock-in a silent BFP expression unit into the AAVS1 (PPP1R12C) safe harbour locus; (2) targeted insertion of Cre recombinase coding sequence to the LMX1A gene immediately upstream of the stop codon (Figure 1A,B). With this design, Cre expression is dependent on that of endogenous LMX1A. Cre/LoxP mediated recombination in LMX1A^+^ cells will lead to the removal of LoxP flanked promoter-less puromycin resistance cassette (PAC-pA) in the AAVS1 locus and hence activate BFP expression. Since genetic PAC-pA removal is inherited, BFP should be expressed in LMX1A^+^ cells as well as their differentiated progeny.

For genome editing in the AAVS1 locus, plasmids containing the AAVS1 donor template and gRNA were electroporated into the KOLF2 cells. A total of 40 puromycin-resistant clones were isolated, expanded, and genotyped by genomic PCR (Figure 1C,D) followed by Sanger sequencing of candidate mutant PCR product. Then, 74% of the clones analyzed had heterozygous BFP insertion (AAVS1^BFP/+)^ while 5% had BFP targeted in both alleles (homozygous BFP insertion, AAVS1^BFP/BFP^, Figure 1E). For Cre knock-in into the LMX1A locus, the LMX1A donor template and gRNA were transfected to one of the homozygous.

BFP lines followed by G418 selection. A total of 54 drug-resistant colonies were picked and expanded clonally. PCR genotyping and Sanger sequencing (Figure 1C,D) identified 15 heterozygous (LMX1A^Cre/+)^ and 5 homozygous (LMX1A^Cre/Cre^) Cre knock-in lines (Figure 1E).

As an initial test for Cre-driven BFP expression, two LMX1A homozygous (LMX1A^Cre/Cre^, AAVS1^BFP/BFP^) and two LMX1A heterozygous lines (LMX1A^Cre/+^, AAVS1^BFP/BFP^) were differentiated towards mDA fate and BFP expression monitored using live cell imaging and Flow cytometry (Figure 1F and Figure S1A,B). At day 30, the LMX1A heterozygous cultures contained 40–60% BFP^+^ cells. In contrast, less than 1% BFP^+^ cells were detected in the LMX1A homozygous cultures (Figure S2B), suggesting that homozygous Cre knock-in in the LMX1A locus may be deleterious to LMX1A expressing cells. Therefore, the two LMX1A^Cre/+^, AAVS1^BFP/BFP^ lines, A17L25 and A17L35, were used for the subsequent studies. For simplicity, these two lines were also referred to as L25 and L35 individually in figures and in general as the LMX1A-Cre/AAVS1-BFP tracer lines. As undifferentiated cells, both cell lines exhibited characteristic pluripotent stem cell morphology and expressed pluripotency markers OCT4, SOX2, and TRA1-81 (Figure 1G,H). Moreover, more than 80% metaphases analyzed were of normal karyotype (46, XY) (Figure 1G) and no new copy number variances were detected compared to the parental KOLF2 iPSC cells (Table S2).

### 3.2. Immunocytochemistry and Flow Cytometry Characterization of LMX1A-Cre/AAVS1-BFP Derived mDA Cultures

**Figure 2:**
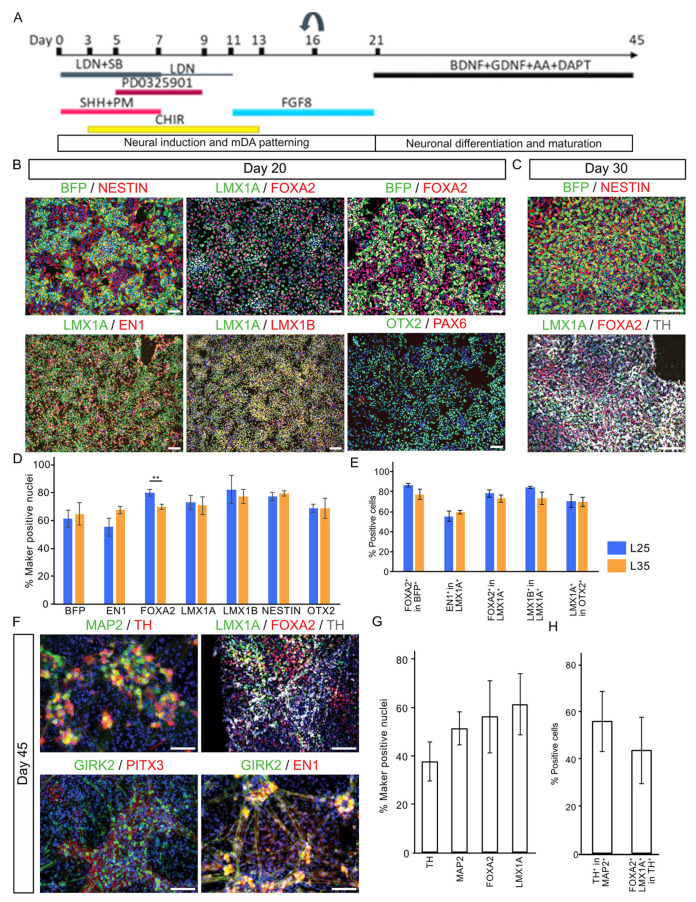
Immunocytochemical characterization of mDA cultures. (**A**) Illustrations of mDA differentiation protocol. (**B**) Shown are representative day 20 cultures stained for antibodies against BFP, NESTIN, LMX1A, FOXA2, EN1, LMX1B, OTX2, and PAX6. (**C**) Day 30 cultures immunostained for BFP, NESTIN, LMX1A, FOXA2, and TH. (**D**,**E**) Bar graph showing quantitative representation of the above, data are presented as mean ± SD of three independent differentiation. Student’s *t*-test was employed for comparison between A17L25 and A17L35 for each measurement. None but FOXA2 was significantly different between the two lines (*p* = 0.009, **). (**F**) Day 45 cultures immunostained for LMX1A, FOXA2, EN1, GIRK2, MAP2, PITX3, and TH. (**G**,**H**) Bar graph showing quantification of day 45 immunostaining. Scale bar 100 μM.

We next examined the mDA differentiation capacity of L25 and L35 lines using a protocol optimized by from Jaeger and Kirks [2,3] followed by immunocytochemical analysis of major mDA lineage markers and the BFP tracer (Figure 2A). At day 20, cultures derived from both cell lines were highly enriched with cells expressing the pan-neural progenitor marker NESTIN and transcription factors expressed in the ventral midbrain and/or playing important roles in mDA fate specification such as LMX1A, FOXA2, OTX2, EN1, and LMX1B (Figure 2B,D) [16,18,19,24,25,26,27]. BFP protein was detected in over 60% of the cells. Around 80–90% of LMX1A^+^ and BFP^+^ cells co-expressed FOXA2, respectively, suggesting a very high correlation between BFP and LMX1A expression (Figure 2E). In contrast, PAX6, a dorsal forebrain marker gene that is also expressed in the lateral midbrain neural progenitors, was barely detected (Figure 2B). These observations confirmed the highly efficient production of ventral midbrain progenitors.

As cultures progressed to day 30, tyrosine hydroxylase (TH) expressing cells became evident, indicating the production of postmitotic DA neurons at this stage (Figure 2C). By day 45, cells expressing other postmitotic mDA neuronal markers (Figure 2F,G), such as PITX3 and GIRK2 were readily detectable while expression of LMX1A and FOXA2 are largely maintained in mDA neurons as demonstrated by co-expression with TH (Figure 2H) [28,29].

**Figure 3:**
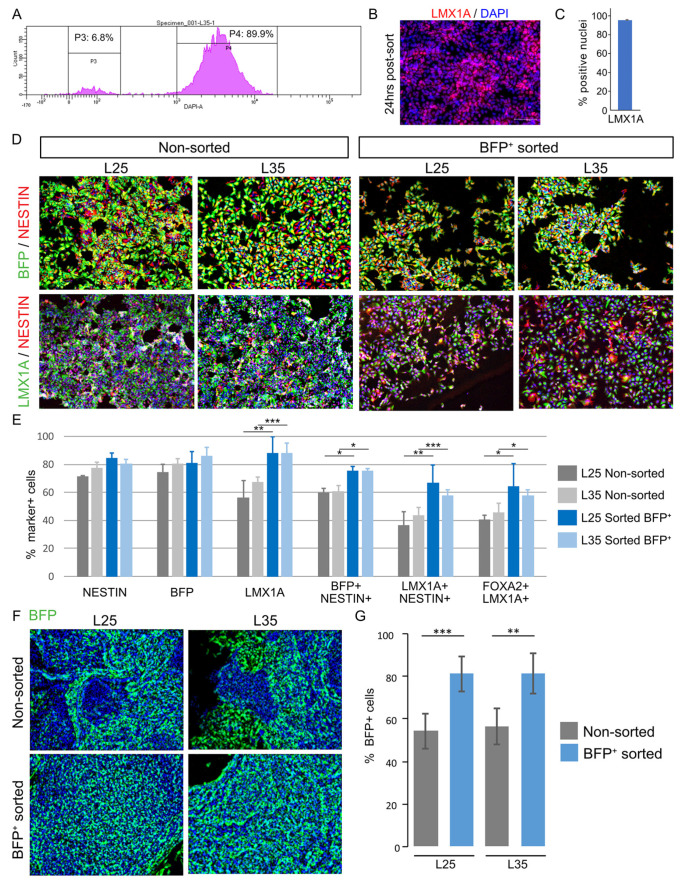
Enrichment of mDA cultures by FACS of BFP^+^ neural progenitors. (**A**) An example of FACS Aria histogram of BFP negative and positive gates for sorting L25 day18 mDA neural progenitors. (**B**) LMX1A antibody staining of sorted BFP^+^ cells 24 h post-FACS. (**C**) Bar graph showing the quantitative representation of the above. (**D**) Representative images of immunostaining for BFP, NESTIN, and LMX1A in non-sorted control and sorted BFP^+^ cultures 3 days post-FACS. (**E**) Bar graphs showing a quantitative representation of the above. (**F**) BFP antibody staining of non-sorted and sorted BFP^+^ cultures 38 days post-FACS (ie. 56 days of total culture time). BFP stained in green, DAPI in blue. Magnification 10x. (**G**) Bar graph showing quantification of the above, Data represent mean ± SD of 3 independent cultures performed with each of the two cell lines. (* *p* ≤ 0.05, ** *p* ≤ 0.01, *** *p* ≤ 0.001).

A prospective application of the LMX1A-Cre/AAVS1-BFP tracer line is the isolation of mDA progenitors and their differentiated derivatives for studies that benefit from a pure population of mDA linage. We therefore tested FACS purification of BFP^+^ cells at day 18 of L25 and L35 differentiation (Figure 3A). Immunostaining for LMX1A showed that 95.40 ± 0.15% of replated sorted BFP^+^ cultures were LMX1A^+^ (Figure 3B,C). Three days after FACS purification, the sorted BFP^+^ cultures contained around 30% more LMX1A^+^ cells than the non-sorted sister control cultures (L25, *p* = 0.005; L35, *p* = 0.001), approximately 10% more NESTIN^+^BFP^+^ (L25 *p* = 0.0316; L35 *p* = 0.013), and 20% increase of NESTIN^+^LMX1A^+^ cells (L25 *p* = 0.005; L35 *p* = 0.001) (Figure 3D,E). At 38 days post-sorting (ie. day 56 of differentiation), we detected a similar proportion of BFP^+^ cells in the sorted BFP^+^ derived cultures compared to that of day 18 (Figure 3F,G) and was approximately 30% higher than that in the non-sorted cultures (L25, *p* = 0.00023; L35 *p* = 0.0032) (Figure 3F,G).

### 3.3. Single-Cell Transcriptomic Analysis Confirms the Generation of Bona Fide mDA Neurons

**Figure 4:**
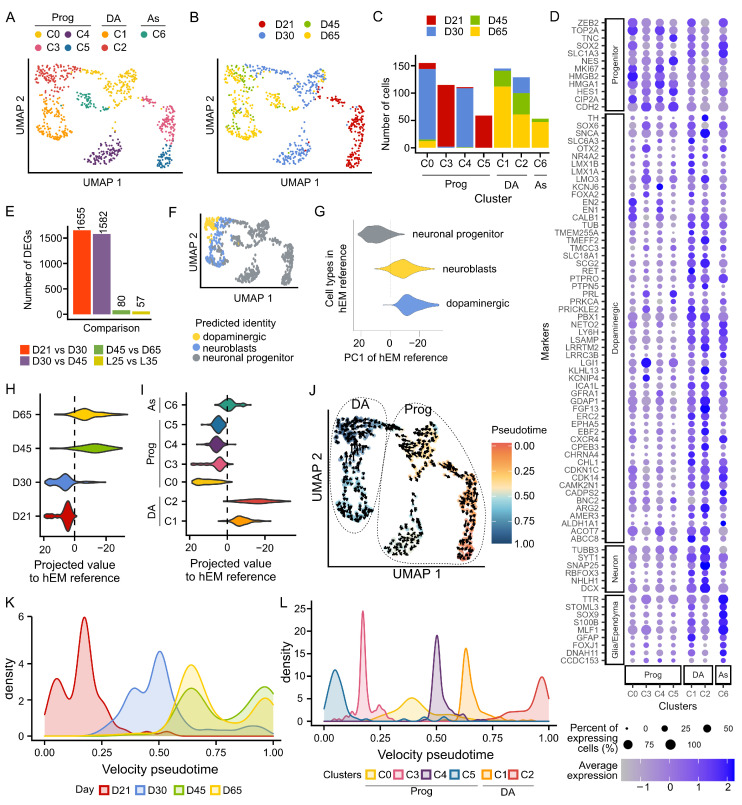
Transcriptional profiling of in vitro differentiation of iPSC into DA neurons. (**A**) UMAP plot colored by the cell clusters identified using the first 10 principal components and the top 5000 most variable features across 767 cells. (**B**) UMAP plot colored by differentiation day showing strong temporal segregation, particularly between d21 and d30. (**C**) Bar graph showing the number of cells per cluster and the distribution of cells from each time point. (**D**) Dot plot of known class-defying markers that aided the annotation of cell clusters (**E**) Bar plot illustrating the number of DEGs between cells of different stages of differentiation and between the two tracer lines. DEGs were identified by a Wilcoxon test, adjusted *p* value < 0.05. (**F**) UMAP shows the predicted cell type using the human embryonic midbrain (hEM) as reference. (**G**) Violin plot illustrating the separation of neuronal progenitors, neuroblasts, and dopaminergic neurons along the first principal component PC1 in the reference developing human midbrain dataset (PMID 27716510). In the hEM, we observed that PC1 segregates progenitors (negative PC1 values) from dopaminergic neurons (positive PC1 values), the violin plot shows the projection of our cells into PC1 grouped by differentiation day in (**H**) and cell cluster in (**I**). (**J**) RNA velocity projected on top of UMAP shows the current and predicted (arrowhead) transcriptional states of cells. Cells are colored by pseudotime, which has been calculated based on RNA velocity across the top 5000 most variable genes and indicates the transition states between cells. (**K**) Density plot shows the distribution of cells by differentiation day along pseudotime. (**L**) Density plot showing the distribution of cells by cluster along pseudotime.

To further evaluate mDA cultures derived from the LMX1A-Cre/AAVS1-BFP tracer lines, we employed the ICELL8 platform and the full-length SMART-Seq2 technology to study the transcriptomic profile of individual cells at days 21, 30, 45, and 65 of differentiation. Of the total 776 qualifying cells (185 at d21, 272 at d30, 78 at d45, and 232 at d65), we detected 13,402 protein-coding genes with an average of 5800 genes per cell. Principal component analysis using the top 5000 most variable genes identified seven unbiased cluster cells (C0 to C6 based on cell numbers, Figure 4A,C). Cells of d21, d30 were strongly segregated from each other and those of d45 and d65, which were clustered closely together (Figure 4B). Accordingly, we detected a greater number of differentially expressed genes (DEGs) between d21 and d30 and between d30 and d45, respectively, compared to DEGs between d45 and d65 (Wilcoxon Test, Padj < 0.05, log2FC > 0.25, Figure 4D,E). Notably, we detected a few DEGs between derivatives of the two tracing lines (Figure 4E and Figure S3A–C), demonstrating a high level of consistency and reproducibility of our lines and differentiation paradigm.

We annotated the cell clusters based on the expression of known gene markers (Figure 4D). C3 and C5 contained mainly d21 progenitor cells and were characterized by high expression of midbrain floor plate marker *CORIN* and basal progenitor marker *HES1*, as well as other early mDA progenitor genes such as *SOX6*, *LMX1A*, *LMX1B*, *OTX2*, *FOXA1*, and *FOXA2* [7,30]. C0 and C4 showed high expression of *TOP2A* that marks mitotic chromosomes and cycling cell marker *MKI67* (Ki67) and a higher expression of mDA progenitor markers *EN1* and *EN2* compared to other clusters. C1, C2, and C6 were made up of d45 and d65 cells, C6 was assigned as an astrocytic lineage due to their expression of *SOX9*, *SLC1A3* (EAAT1), and *S100B*. C1 and C2 were characterized as DA neurons based on their broad expression of neuronal gene *SYT1* in addition to several dopaminergic markers including *TH*, *CALB1*, *RET*, *PBX1*, *SLC18A1* (VMAT1), and *SLC6A3* (DAT). However, C2 shows a highly enriched expression of SNAP25, suggesting a more mature neuronal state. To corroborate the cluster annotation, we analyzed our data using the transcriptional profile of ‘neuronal progenitors’, ‘neuroblast’, and ‘dopaminergic neurons’ from the human embryonic midbrain (hEM) as reference [31]. Indeed, our progenitor clusters (C0, 3, 4, and 5), were predicted as neuronal progenitors while C1 and C2 were mapped to a mix of neuroblasts and dopaminergic neurons (Figure 4F and Figure S3F).

We noticed that the first principal component (PC1) of the hEM separated neuronal progenitors (positive PC1 values) from neuroblasts and dopaminergic neurons (negative PC1 values, Figure 4G). Thus, we projected our scRNA-seq time course gene expression data into the same PC1 and observed a strong segregation of d21 and d30 cells from d45 and d65 cells, which projects closer to where hEM neuroblasts and dopaminergic neurons lie (Figure 4H). PC1 projection also supports the more mature nature of C2 compared to C1 (Figure 4I). Finally, we calculated the RNA velocity of these cells, a method that examines the ratio of unspliced/spliced mRNA for predicting the future state of individual cells and hence the directionality of the differentiation trajectory (Figure 4J) [31]. Applying RNA velocity to individual cells within C0-5, we observed even better segregation between cells of differentiation of d45 and d65 cells from d21 or d30 cells (Figure 4K). Consistent with principal component projection and reference mapping results, RNA velocity trajectory analysis demonstrates that C2 is more mature than C1 and other progenitor populations (Figure 4L).

Taken together, our scRNAseq analysis confirmed the efficient generation of ventral midbrain progenitors and postmitotic mDA neurons using the optimized differentiation paradigm. Moreover, d45 mDA neurons exhibit similar transcriptomic profiles to those at d65.

### 3.4. Preferential Sensitivity of BFP^+^ Neurons to MPP^+^ Toxicity

**Figure 5:**
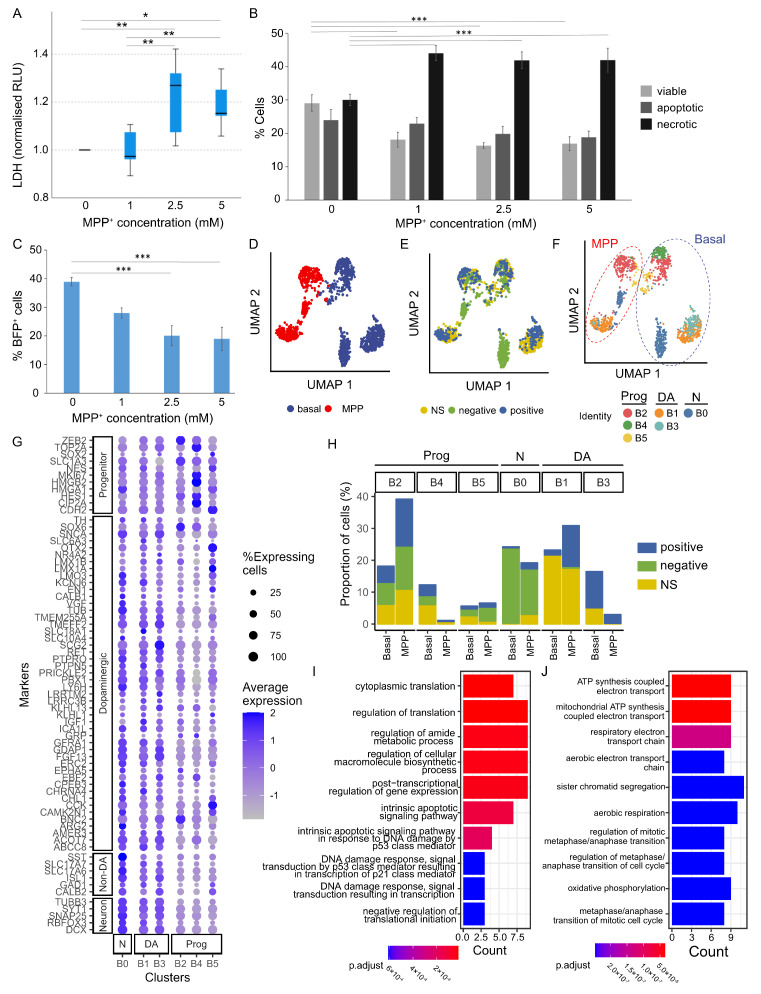
Modelling PD-related toxicity using MPP^+^. (**A**) Quantification of MPP^+^ cytotoxicity by LDH assay. Kruskal–Wallis H Test, *p* = 0.003, Pairwise comparisons: 0 mM vs. 1 mM, *p* = 0.797, 0 mM vs. 2.5 mM, *p* = 0.008, 0 mM vs. 5 mM, *p* = 0.020, 1 mM vs. 2.5 mM, *p* = 0.004, 1 mM vs. 5 mM, *p* = 0.010, 2.5 mM vs. 5 mM, *p* = 0.838. (**B**) Quantification of viable, apoptotic, and necrotic cells determined by Annexin V assay. ANOVA, *p* = 0.001 for viable cells; *p* = 0.009 for necrotic fraction. (**C**) Increasing doses of MPP^+^ led to progressive decrease in BFP^+^ cells. ANOVA, *p* = 0.001. Data shown in (**A**–**C**) represents mean ± s.e.m. from 3 biological replicates each with 3 technical replicates per condition of two independent cell lines. * *p* ≤ 0.05, ** *p* ≤ 0.01, *** *p* ≤ 0.001. (**D**) UMAP plot showing strong segregation of untreated (basal) and MPP^+^ treated cells (a total of 1418 cells). (**E**) UMAP plot including basal and MPP^+^ treated cells colored by sorted status (BFP^+^, BFP^−^ and NS). (**F**) UMAP plot colored by clusters identified for the basal cells (B0 to B5) and predicted clusters of MPP^+^ treated cells using the basal cell profile as reference. (**G**) Dot plot showing the expression of marker genes in different clusters of both basal and MPP^+^-treated cells. (**H**) Bar graph showing the distribution and portion of cells in each of the 6 clusters, which are presented separated by the sorting status. (**I**,**J**) Top 10 gene ontologies (GO) enriched in the DEGs upregulated (**I**) and downregulated (**J**) in MPP^+^ treated B3 population compared to basal B3 population.

1-Methyl-4-phenyl-1,2,5,6-tetrahydropyridine (MPTP) or its metabolite methyl-4-phenylpyridinium (MPP^+^) are known to selectively damage mDA neurons and produce symptoms that resemble those observed in Parkinson’s disease (PD). To demonstrate the relevance of our iPSC-derived mDA neurons in PD modelling, we tested MPP^+^ cytotoxicity to d45 cells by quantifying the release of lactate dehydrogenase (LDH) to the culture media as a measure of cell death. After 24 h treatment with or without 1, 2.5, and 5 mM MPP^+^, we detected a significant increase of LDH in cultures treated with 2.5 and 5 mM MPP^+^ (Figure 5A). To complement the above, we performed a cytometry-based Annexin V binding assay. Combined with propidium Iodide (PI) staining, this assay distinguishes live, dead (necrotic), and apoptotic cells. We found a significant decrease in the proportions of viable cells (*p* = 0.001), representing approximately a 35% reduction of total live cells in the 2.5 and 5 mM MPP^+^ treatment conditions. Concurrent with the decreased number of live cells, we observed an increase in the proportions of necrotic cells (*p* = 0.009) across all MPP^+^ doses in comparison with the vehicle controls (Figure 5B). Interestingly, parallel quantification of BFP^+^ cells of these cultures revealed a progressive decrease in this population with increasing MPP^+^ dosage (*p* = 0.001) (Figure 5C). The ~50% reductions of BFP^+^ cells under 2.5 and 5 mM MPP^+^ conditions were greater than that of total live cells in the same treated cultures, suggesting that BFP^+^ neurons (ie. derivative of LMX1A neural progenitors) are preferentially vulnerable to MPP^+^ toxicity.

To substantiate the above findings, we performed scRNAseq analysis on d45 cells treated with or without 1 mM MPP^+^ for 24 h. Both the vehicle control (basal) and MPP^+^ treated groups included non-sorted (NS) d45 cells and d45 cells derived from d18 sorted BFP^+^ and BFP^−^ cell fractions. The basal and MPP^+^ treated cells were clearly segregated (Figure 5D), while segregation by sorting status was less prominent overall except for populations of d18 sorted BFP^−^ fraction (Figure 5E). We first identified six clusters among all cells from the basal condition samples, which segregated mainly into progenitors (B2, B4, B5) and neurons (B0, B1, B3, Figure 5F,G and Figure S4A). We observed segregation by their sorting status such that B3 was largely composed of derivatives of sorted BFP^+^ cells whereas B0 was mainly made up of BFP^−^ fraction (Figure 5F and Figure S4A,B). B1 and B3 cells exhibit mDA transcriptomics characteristics, with B3 cells more mature than B1 as exemplified by their richer expression of *SNAP25* and *SYT1* (Figure 5G). In contrast, the B0 cluster expresses higher levels of or contains a greater proportion of cells within the cluster that express genes not compatible with DA neurons (e.g., *STT*, *SLC17A6/7*, *ISL1*). Using the basal clusters as a reference, we next predicted the identity of MPP^+^ treated cells (Figure 5F and Figure S4C–E) and compared the proportion of each cluster between the basal and MPP^+^ conditions using a permutation test (Figure 5F and Figure S5). Among the neuronal clusters, only the B3 DA cluster showed a significant reduction in the after MPP^+^ exposure (FDR < 0.001998), implying preferential sensitivity of B3 neurons.

We next performed differential expression analysis in the neuronal B3 population between the MPP^+^ and basal conditions. Despite the small number of B3 cells obtained following the MPP^+^ treated condition, we were able to identify 78 DEGs (adjusted *p* value < 0.05, Supplementary Tables S4–S6). Gene Ontology enrichment analyses were separately performed with the upregulated (n = 28) and downregulated genes (n = 50). The biological pathways enriched in the upregulated genes include intrinsic apoptotic signaling while downregulated pathways include oxidative phosphorylation, consistent with the mitochondrial toxicity of MPP^+^ and also implicated role of one-carbon metabolism in neurodegeneration [32,33] (Figure 5I,J). Together, our transcriptomic analysis provides support that a fraction of BFP^+^ neurons are particularly sensitive to MPP+ cytotoxicity.

## 4. Discussion

Using CRISPR/Cas9 assisted knock-in of Cre and BFP in the LMX1A and AAVS1 loci, respectively, we successfully generated LMX1A-Cre/AAVS1-BFP tracer lines for labelling hiPSC-derived LMX1A^+^ mDA neural progenitors and their differentiated neuronal progenies. We showed, via immunocytochemical analysis and single-cell transcriptomic profiling, that BFP^+^ cells co-expressed LMX1A and cardinal mDA neural progenitor markers at the neural progenitor stage of in vitro differentiation, which progress into postmitotic TH^+^ dopamine neurons as differentiation progressed. The mDA progenitors were amenable to FACS purification and subsequent differentiation into postmitotic dopaminergic neurons.

Initially demonstrated in mouse ESCs via knocking GFP into the Pitx3 locus [34], a number of PSC lines have been reported to date for sorting DA neurons. These sorting systems are based on the targeted insertion of a reporter into a gene with a restricted expression pattern in the mDA lineage (PITX3 or LMX1A) [35,36] or expressed in all DA neurons (eg. TH) [37]. The reporters in these tools mirror the expression of their target genes in real time so cells isolated using these systems are target gene-expressing cells at the time of sorting. The LMX1A-Cre/AAVS1-BFP tracer line reported here differs from those tools in that BFP is expressed in cells expressing LMX1A at the time of analysis as well as progeny of LMX1A^+^ cells that themselves may no longer be expressing LMX1A. Since LMX1A expression is downregulated in a subpopulation of mDA neurons and does not cover the entire mDA neuronal population [31,38], the LMX1A-Cre/AAVS1-BFP line would have an advantage over the LMX1A-GFP knock-in line for isolating postmitotic mDA neurons, as the PITX3-GFP system. Another application of the LMX1A tracing system that cannot be fulfilled by a postmitotic reporter system, such as PITX3- or TH-based knock-in, is for lineage tracing studies. For example, to investigate potential regional characteristics of glial cells derived from LMX1A^+^ floor plate progenitors.

Several cell surface markers have been identified for isolating ventral midbrain neural progenitors, which include CORIN [17], TPBG [39]; LRTM1 [40] amongst others [41,42,43]. Cell surface marker-based purification has advantages over the genetic-based approach as it can be readily applied to all PSC lines. It was reported that TPBG^+^ cells contain ~50% of LMXA^+^FOXA2^+^ cells while LRTM1 sorting enriched LMX1A^+^FOXA2^+^ cells to 77.2 ± 2.1%. Thus, new cell surface markers may be needed to cover the whole spectrum of mDA progenitors.

Our scRNAseq profiling of d21-65 cultures and integrated analysis with that of the human fetal ventral midbrain demonstrates that our iPSC-derived cell populations corresponded very well to progenitors and dopaminergic neurons of the hEM [31], providing unbiased confirmation of their mDA progenitor and neuronal identities. Interestingly, while morphological maturation continued to progress in culture from d45 onwards, we found that d45 and d65 cells were very similar at the transcriptional level. This finding suggests that for studies that primarily measure transcriptomics changes, for instance, high throughput drug or gene screening via scRNAseq, one could save 20 days in vitro culture by using d45 mDA neurons.

By modelling PD-related drug toxicity using MPP^+^, we found that the dose-dependent increase of cell death and decrease of total live cell cells is accompanied by a greater reduction in the proportion of BFP^+^ neurons within the same MPP^+^ treated cultures, hence demonstrating preferential susceptibility of BFP^+^ neurons to MPP^+^ toxicity. Interestingly, single-cell transcriptomic analysis of MPP^+^ treated neurons derived from d18 sorted BFP^+^ and BFP^−^ neural progenitors and non-sorted sister cultures revealed the biggest degree of cell loss being the B3 cluster, which contains mostly of d18 sorted BFP^+^ fraction with essentially no contribution from the sorted BFP^−^ fraction. Together, these findings strongly support the relevance of using BFP^+^ neurons for studying PD-related damage and vulnerability.

Attempts were made to provide insights into potential transcriptomics characteristics of MPP^+^ sensitive B3 neurons by comparing gene expression between the basal and MPP^+^ treated B3 cells. This had proved challenging as it was difficult to discern whether the DGEs detected were driven by cell identity or from their response to MPP^+^. Further, scRNAseq with greater number of cells at basal and MPP^+^ treated conditions may help to reveal heterogeneity within the B3 population and allow the identification of BFP^+^ sensitive subpopulations and their molecular characteristics without compounding effects of MPP^+^ regulated gene expression.^.^

## 5. Conclusions

In summary, this work established a new tool to study human midbrain dopaminergic neuron development and a more refined model to study PD-related toxicity and investigations into PD etiology in combinations with CRISPR/CAS9 genome editing of associated risk genes. As a tracer line, the LMX1A-Cre/AAVS1-BFP iPSC cells can provide a unique opportunity for investigations into differentiated derivatives of midbrain floor plate progenitors beyond mDA neurons.

## Data Availability

The sequencing data discussed in this publication have been deposited in NCBI’s Gene Expression Omnibus [44] and are accessible through GEO Series accession number GSE247600 and GSE249360. Code is available at GitHub (https://github.com/jmonzon87/LMX1A_iPSC_DA, accessed on 8 November 2023).

## Supplementary Materials

The following supporting information can be downloaded at: https://www.mdpi.com/article/10.3390/cells12242860/s1, Supplementary methods, Figure S1: Flow cytometry analysis for BFP expression; Figure S2: Gating strategy used for Annexin V and PI staining by Flow cytometry; Figure S3: Characterization of gene clusters during mDA differentiation; Figure S4: Effects of MPP^+^ to cells in different clusters; Figure S5: Proportion difference per cluster in basal and MPP^+^ conditions; Table S1: PCR primers; Table S2: CNV analysis of the parental and engineered iPSC lines; Table S3: Primary antibodies used; Table S4: DEGs comparing MPP^+^-treated B3 mDA neurons with untreated.; Table S5: Gene Ontology enrichment of DEGs upregulated in MPP^+^-treated B3 mDA neurons.; Table S6: Gene Ontology enrichment of DEGs downregulated in MPP^+^-treated B3 mDA neurons.
