# Supplementary material for: Single-Cell Transcriptomics and In Vitro Lineage Tracing Reveals Differential Susceptibility of Human iPSC-Derived Midbrain Dopaminergic Neurons in a Cellular Model of Parkinson’s Disease
Source: Cells. 2023 Dec 18;12(24):2860. doi: 10.3390/cells12242860 (PMC10741976; doi:10.3390/cells12242860)
Supplement: Supplementary file 1 [file cells-12-02860-s001.zip › Supplementary Materials.docx]

**Supplemental Methods**

## CRISPR/Cas9 genome editing of human iPSCs

Two rounds of CRISPR/Cas9 genome editing were carried out to generate the LMX1A^Cre^-BFP tracer lines. The first round of editing concerns targeted insertion of a BFP expression cassette downstream of loxP flanked puromycin expression unit in the *AAVS1* safe harbor locus. In the second round genome editing, a CRE protein expression cassette was knocked-in into the LMX1A locus. This strategy allows LMX1A dependent expression of Cre, which then removes the floxed puromycin cassette sandwiched between the CAG promoter and BFP-poly A sequence in the *AAVS1* locus, leading to LMX1A controlled expression of BFP (Fig. 1). Detailed description of CRISPR/Cas9 editing and associated genotyping is provided in Supplemental methods.

In order to target LMX1A 3’UTR, two guide RNAs (gRNAs) were designed comparing two independent CRISPR gRNA design tools: Atum CRISPR gRNA (former DNA2.0 <https://www.atum.bio/eCommerce/cas9/input>) and the CRISPR Design Tool ([http://crispr.mit.edu](http://crispr.mit.edu/)) to minimize the risk of off-target effects of Cas9 nuclease ([Figure](http://diabetes.diabetesjournals.org/content/64/7/2497.long#F1) 1a). gRNA1 5′- GCCTAGTCACAGAACTCTAGGGG, gRNA2 5′-TCTGTGACTAGGCTCCCATATGG. All gRNAs were synthesized as oligonucleotides and cloned into the px330A1x2 plasmid (Addgene) following the protocol of Sakuma et al. 2014 (XX). A donor template (gene targeting) vector for homologous recombination was constructed that contains a P2A-CRE-pA and a CAG-Neo-pA selection cassette flanked by 600 bp 5’ and 3’ homologous arms (HA). The gRNA target sites are located within the 4336 bp region between the two homologous arms (Figure 1).

In order to target PPP1R12C intron 1 (AAVS1 safe harbor), two guide RNAs (gRNAs) were designed comparing two independent CRISPR gRNA design tools: Atum CRISPR gRNA (former DNA2.0 <https://www.atum.bio/eCommerce/cas9/input>) and the CRISPR Design Tool ([http://crispr.mit.edu](http://crispr.mit.edu/)) to minimize the risk of off-target effects of Cas9 nuclease ([Figure](http://diabetes.diabetesjournals.org/content/64/7/2497.long#F1) 1a). gRNA1 5′- GTTAATGTGGCTCTGGTTCTGGG, gRNA2 5′- ACCCCACAGTGGGGCCACTAGGG. All gRNAs were synthesized as oligonucleotides and cloned into the px330A1x2 plasmid (Addgene) following the protocol of Sakuma et al. 2014 (XX). A donor template (gene targeting) vector for homologous recombination was constructed that contains the TagBFP gene and a selection cassette: CAG-loxP-Puro-pA-loxP-TagBFP-pA flanked by 756 bp 5’HA and 837 bp 3’HA. The gRNA target sites are located within the 4794 bp region between the two homologous arms (Figure 1).

To Knock-in the AAVS1 locus, KOLF2-C1 cells were transfected with a total of 5ug DNA in a ratio 2:3 (gRNAs:donor template) and 7ug DNA in a ratio 2:5 using the Amaxa P3 Primary Cell 4D-Nucleofector Kit (Lonza) with pulse condition CB-137. Puromycin was added 48h after electroporation at a concentration of 0.25ug/ml and increased to 0.5ug/ml 2 days later. After a week, colonies were dissociated with Accutase and single cells plated at 2000 cells density into 10cm dishes. Individual drug resistant colonies (n=40) were manually picked one week later and expanded clonally. Genotyping was done by genomic PCR (primers used are provided in **Table S1**) followed by Sanger sequencing of candidate mutant PCR product. One of the homozygous lines was used for a second round of targeting to knock-in CRE gene in the LMX1A locus. KOLF2-C1 BFP^+/+^ cells were transfected with a total of 5ug DNA in a ratio 1:4 (gRNAs:donor template) using the Amaxa P3 Primary Cell 4D-Nucleofector Kit (Lonza) with pulse condition CB-137. G418 was added 48h after electroporation at a concentration of 25ug/ml and gradually increased to 100ug/ml. After a week, colonies were dissociated with Accutase and single cells plated at 2000 cells density into 10cm dishes. Individual drug resistant colonies (n=54) were manually picked one week later and expanded clonally. Genotyping was done by genomic PCR (primers used are provided in Table S1) followed by Sanger sequencing of candidate mutant PCR product.

Two of the KOLF2-C1 BFP^+/+^ CRE^+/-^ and two of the KOLF2-C1 BFP^+/+^ CRE^+/+^ derived lines were used in the present study.

Sub-confluent PSCs cultures were treated with 0.1μg/ml Demecolcine (Sigma D1925) for 1 hour at 37 °C and then dissociated to a single cell suspension using Accutase (ThermoFisher) for 10 minutes at 37 ^o^C. Cells were collected, washed twice with PBS by centrifugation for 4 min at 900 rpm, resuspended in 2 ml of PBS and 6 ml of 0.075 M KCl hypotonic solution was added to the tubes following incubation at 37 °C 15 min. Additional 4 ml of 0.075 KCl was added after incubation and cells were collected by centrifugation for 4 min at 900 rpm. The supernatant was removed leaving 300 μl to resuspend the cell pellet by flicking. 4 ml of pre-chilled (-20 °C) methanol/acetic acid (3:1, VWR chemicals) was added dropwise and flicking to homogenize. Cell suspension was incubated for 30 min at room temperature. Cells were then centrifuged for 4 minutes at 900 rpm and resuspended with additional 4 ml of methanol/acetic acid. Cells were collected as previously and resuspended in 300 μl of methanol/acetic acid. Cell suspension was dropped onto a slide (pre-chilled and laid on angle) from a height of around 30 cm. Slides were air dried and chromosome spread was stained and mounted using a mix of mounting media with DAPI (1:3000). Images of chromosome spreads were obtained with an inverted microscope. Images were acquired at 100x using the Leica Application Suite software and manually counted on ImageJ.

**Karyotyping**

Sub-confluent iPSCs cultures were treated with 0.1μg/ml Demecolcine (Sigma D1925) for 1 h at 37 °C and then dissociated to a single cell suspension using Accutase (ThermoFisher) for 10 min at 37^o^C. Cells were collected and washed twice with PBS by centrifugation for 4 min at 900 rpm. Cells were resuspended in 2 ml of PBS and 6 ml of 0.075 M KCl hypotonic solution was added to the tubes following incubation at 37 °C 15 min. Additional 4 ml of 0.075 KCl was added after incubation and cells were collected by centrifugation for 4 min at 900 rpm. The supernatant was removed leaving 300 μl to resuspend the cell pellet by ficking. 4 ml of pre-chilled (−20 °C) methanol/acetic acid (3:1, VWR chemicals) was added dropwise and ficking to homogenize. Cell suspension was incubated for 30 min at room temperature. Cells were then centrifuged for 4 min at 900 rpm and resuspended with additional 4 ml of methanol/acetic acid. Cells were collected as previously and resuspended in 300 μl of methanol/acetic acid. Cell suspension was dropped onto a slide (pre-chilled and laid on angle) from a height of around 30 cm. Slides were air dried and chromosome spread was stained and mounted using a mix of mounting media with DAPI (1:3000). Images of chromosome spreads were obtained with an inverted microscope. Images were acquired at 100×using the Leica Application Suite software and manually counted on ImageJ.

**Genomic integrity screen**

The genomic integrity of the two lines used in this study and the parental Kolf2 line were analysed using the Infinium Global Screening Array v2.0 (Illumina). The data were uploaded to the Illumina Genome Studio v2.0.4 and call rate threshold set at 0.95. Datasets were analysed using the PennCNV software (<http://penncnv.openbioinformatics.org/en/latest/>) with GrCh38/hg38 as reference assembly. Quality controls steps included merging of CNVs with between CNV fraction of <0.2 (<20%), and exclusion of those <100 000 bp in length and containing <10 single nucleotide polymorphisms (SNPs). Log R ratio and B allele frequency plots were generated for each CNV and checked for reliability of the call.

## Methods associated with single cell transcriptomics analysis

Single cell RNA library generation and sequencing

We prepared single cell preparations from fresh cultures by incubation with Accutase 10 min at 37C followed by gently pipetting the cells 3-4 times and an extra 2 min incubation at 37C. After dissociation and pelleting, cells were pipetted to single cells in 500 ul PBS and incubated for staining with Hoechst and PI for 20 min at 37C (ReadyProbes™ Cell Viability Imaging Kit, Blue/Red, R37610 Invitrogen). After washing, we re-suspended the cells in 1ml PBS and filtered through a nylon membrane to remove cell clumps. We used Countess II Automated cell counter (Invitrogen) to quantify cell numbers and cells were dispensed at a concentration of 30.000 cells/ml into ICELL8 Single-Cell System (Takara). We followed the SMART-Seq ICELL8 cx Application Kit protocol to generate the libraries.

Quality control performed using the Qubit dsDNA HS Assay Kit (Q32851, Thermo Fisher Scientific) in a Qubit fluorometer, and the DNF-474 High sensitivity NGS analysis kit in the Fragment Analyzer (Agilent).

Sequencing of time course library carried out on Illumina HiSeq 4000, using 75 base paired end sequencing with dual indexing. Sequencing of basal and MPP+ treated cultures library carried out on the Illumina NovaSeq 6000 platform, using 100 base paired end sequencing with dual indexing.

## scRNA-seq data processing

For initial quality control we ran *FastQC* and summarized reports with *MultiQC*. We followed the Mappa analysis pipeline from Takara (v 1.0). Briefly, we ran *demux* for demultiplexing and barcode assignment followed by *analyzer* to obtain gene counts (Consists of *Cutadapt* for adapter removal and trimming, *STAR* for genome alignment, *Subread* for counting and custom scripts for summarization). We generated a custom genome index (STAR-2.7.3a) including the human reference (hg38, with gene annotations from Ensembl release 99) and the BFP sequence using default parameters (except for –sjdbOverhang = 75, and ---limitGenomeGenerateRAM = 168633417088).

## scRNA-seq data filtering

We filtered gene expression to keep only protein coding genes. We removed cells with particularly low number of genes detected (< 2000), and cells with extremely high percentage of the library mapping to mitochondrial genes (> 20%). Also, we removed lowly expressed genes keeping only those with more than 1 count in at least 5% of filtered cells. For the in vitro iPSC to DA differentiation time course experiment, a mean of ~1.1M mapped reads allowed us to detect a mean of ~5.8K protein coding genes per cell. For the cell populations where we compared of NS, BFP positive and BFP negative cells as well as the MPP+ treatment (24h) we additionally removed cells with low number of reads < 100K.

## Dimensionality reduction and clustering

Gene expression data for the in vitro differentiation time course was log normalized and a scaling factor of 1x10^6^. Principal component analysis (PCA) was based on the top 5000 most variable features obtained with the *FindVariableFeatures* using the vst selection method implemented in Seurat. UMAP was run based on the first 10 PCs. For cell clustering we used the shared nearest neighbour (SNN) modularity optimization algorithm (Modularity Optimizer version 1.3.0 by Ludo Waltman and Nees Jan van Eck), and a resolution of 0.5 for cluster identification.

For the second scRNA-seq gene expression dataset (sorted/unsorted/MPP+ treatment), we normalized gene expression data separately samples from Plate A (sorted populations and MPP at 24h) and B (MPP time course response). For plate A, we performed PCA separately on the basal and MPP+ treated cells. As before, we selected the top 5000 most variable genes and ran UMAP using the top 10 PCs, but with a resolution of 1 and 1.5 for the basal and MPP+ subsets respectively.

## Differential Expression Analysis

To identify gene markers and contrast experimental groups we used a Wilcox test implemented in the *FindMarkers* function from Seurat R package. Differentially expressed genes (DEGs) were defined as those with an adjusted p value < 0.05, log Fold change > 0.25. For cluster markers, where cells within a cluster are compared to the rest, we only considered positive markers.

## Gene Ontology enrichment analysis

## We used *ClusterProfileR* for Gene Ontology (GO) enrichment analysis, using the respective background gene population of each filtered dataset. We adjusted for multiple testing using the Benjamini and Hochberg correction method, GO terms with an adjusted p value < 0.05 were considered enriched. Enriched GO terms were simplified based on semantic similarity using Revigo (SimRel method and threshold of 0.5, PMID: 21789182).

## Use of the human embryonic midbrain as reference

Molecule counts for the human embryonic midbrain (hEM, GSE76381) were accessed through GEO. We subset gene expression data of neuron progenitors, neuroblasts and dopaminergic neurons. We only kept genes shared the hEM and the in vitro time course of iPSC to DA neuron differentiation (n = 12309 genes). Counts in both datasets were normalized separately, using vst normalization method and a scale factor of 1x10^6^. The top 5000 most variable features in the hEM were used to perform PCA. We projected the iPSC-DA dataset onto the PCs of the hEM using matrix multiplication, the scaled and centred gene expression data from the iPSC-DA dataset multiplied by the gene loadings of the hEM. Also, we used the hEM dataset to aid the annotation of our cell clusters. First, we carried out the integration of the hEM dataset and our time course iPSC to DA neuron differentiation. Anchors were based on the first 10 PCs, and prediction of cell type was done using the hEM as reference using the *TransferData* function from Seurat [PMID: 31178118].

RNA velocity

RNA-velocity analysis was carried out using the counts including introns from the Mappa (1.0) pipeline to infer intron and exon counts as proxy of un-spliced and spliced RNAs. Spliced counts were log transformed and normalized with the *logNormCounts* function in scuttle R library. The top 5000 most variable features were identified based on the residual of the mean and variance fit according to the *modelGeneVar* and *getTopHVGs* functions from the scran R library. RNA velocity was estimated based on the most variable features using the scvelo function from the velociraptor library based on scVelo in Python. The velocity vector was then projected onto the UMAP embedding using the *embedVelocity* function. The astrocyte cluster C6 was excluded in this analysis.

Cell proportion test

For the MPP^+^ dataset, we identified cell clusters in the basal cells and used these as reference to predict the cell cluster identity of the MPP^+^ treated cells. We tested the change in proportion between basal and MPP^+^ cell clusters using a randomization test as implemented in scProportionTest (PMID: 34035083), in which labels from both samples were mixed and 10000 random samples were used to estimate the expected proportion difference. A numeric p value is drawn from the randomization and corrected for multiple testing using FDR.

## Supplemental Figures


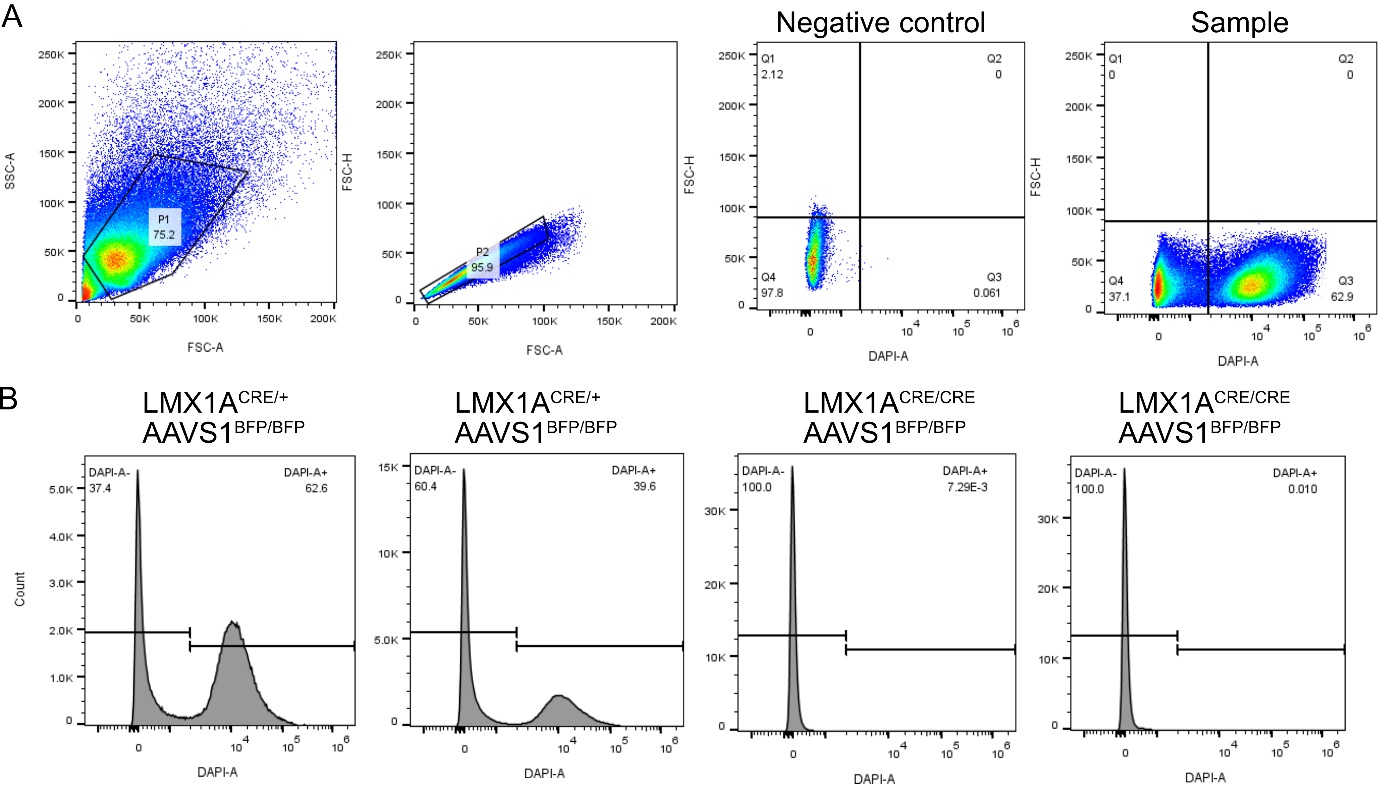


**Figure S1.** Flow cytometry analysis for BFP expression. (A) Undifferentiated LMX1A-Cre/AAVS1-BFP line was used as a negative control for gating BFP negative cells. (B) Flow cytometry analysis of day 30 mDA differentiation of two LMX1A^Cre/+^ and LMX1A^Cre/Cre^ lines, respectively, revealing negative effect of Cre homozygous knock-in on BFP expression.


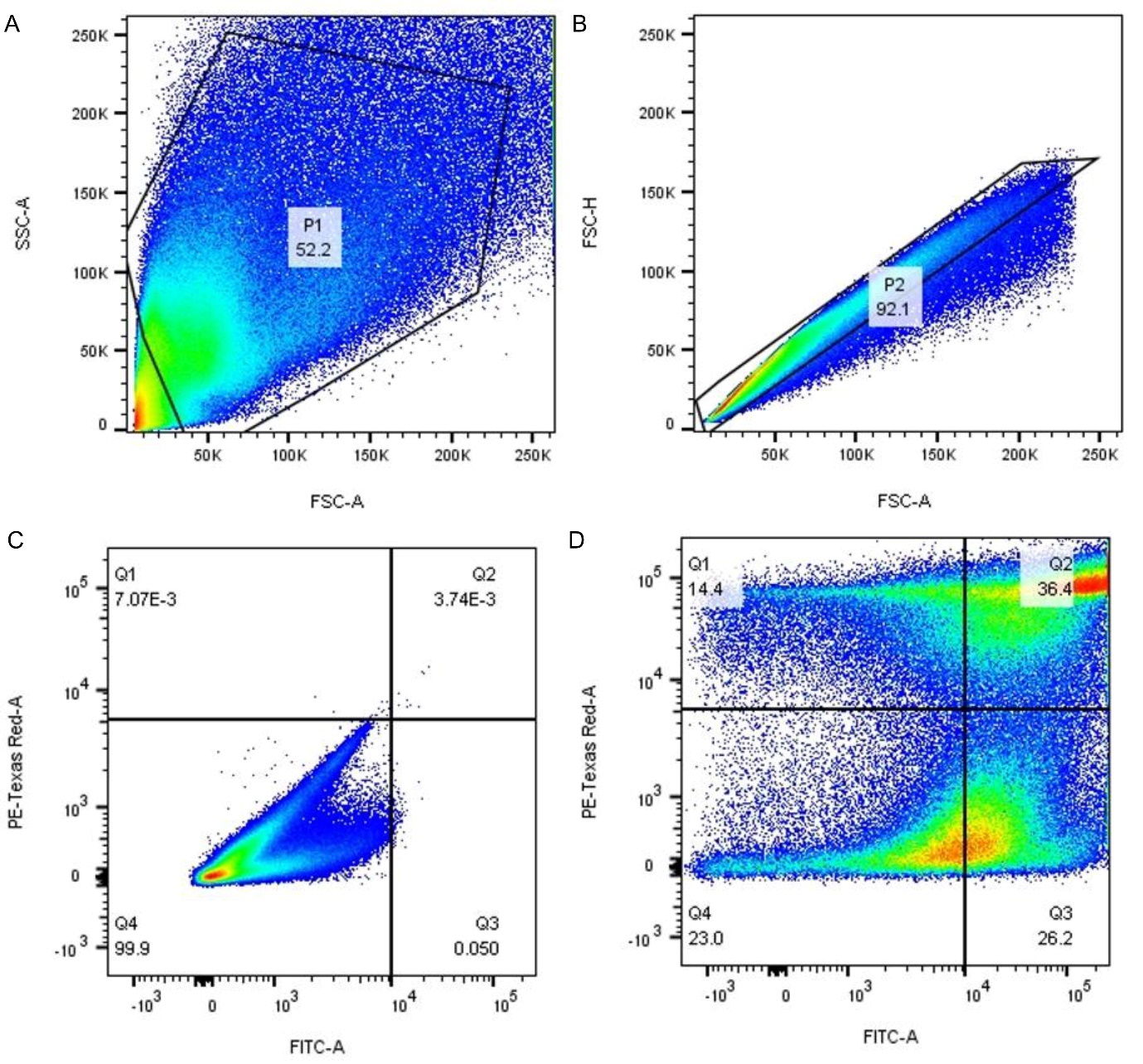


**Figure S2.** Gating strategy used for Annexin V and PI staining by Flow cytometry. (A) P1 snapshot. (B) P2 or single cell population snapshot. (C-D) Cell population distribution in quadrants: Q4-alive, Q3-apoptotic, Q2-necrotic. Annexin V in FICT-A channel and PI in PE-Texas red channel. Example of sample treated with vehicle (C), and MPP^+^ (D).

**
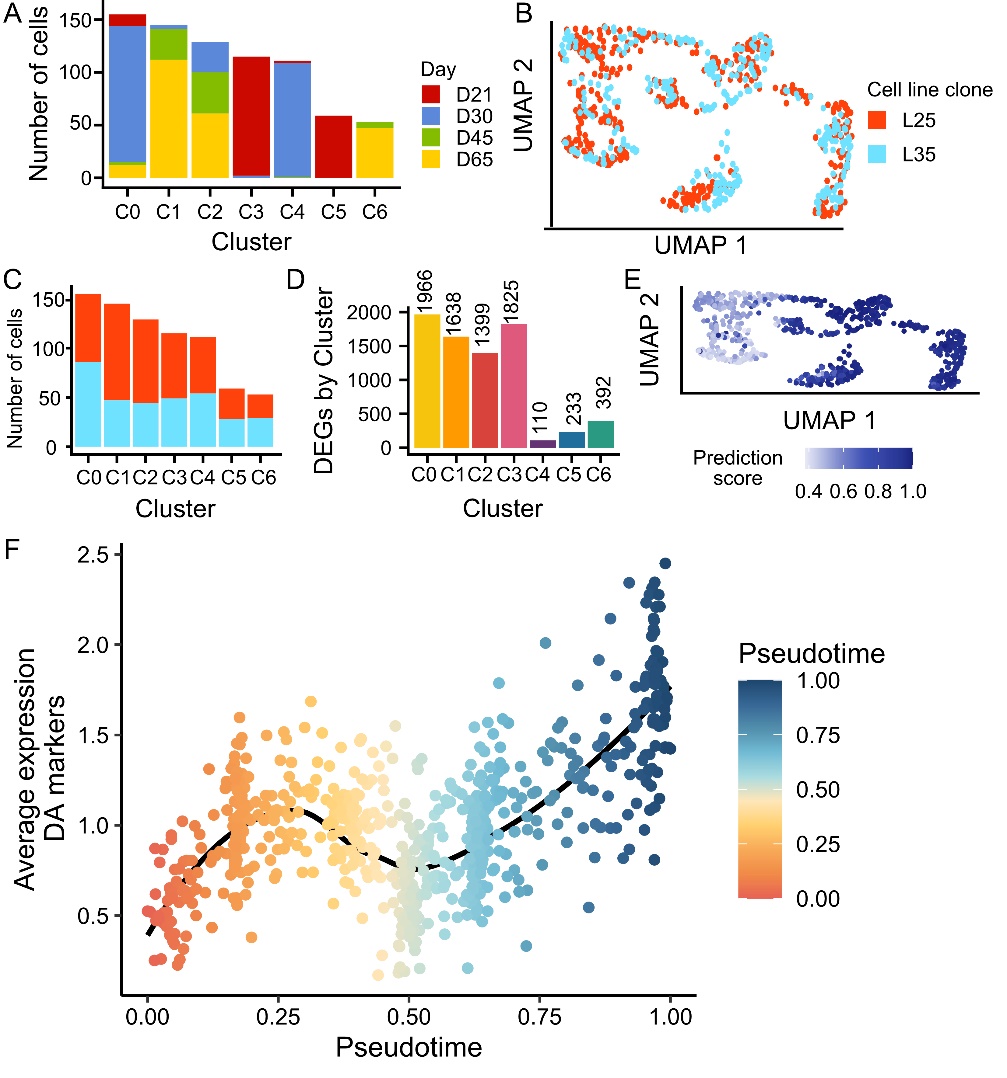
**

**Figure S3.** Characterization of gene clusters during mDA differentiation (A) Bar graph showing the distribution of time course samples in each cell cluster. (B) UMAP plot of all time course samples coloured by clone. (C) Bar graph shows the number of cells per cluster corresponding to each clone. (D) Number of differentially expressed genes that characterize each cell cluster compared to all other clusters (Wilcoxon test, Padj< 0.05). (E) iPSC derived cells were reference mapped to neuronal progenitors, neuroblasts and dopaminergic neurons to a published human embryonic midbrain scRNAseq data (PMID 27716510). UMAP plot coloured by the prediction score of this integration. Prediction score ranges from 0 to 1, indicating low (0) and high (1) confidence in the predicted label/cell type. (F) Average expression of DA markers along the pseudotime trajectory shown in Figure 4.


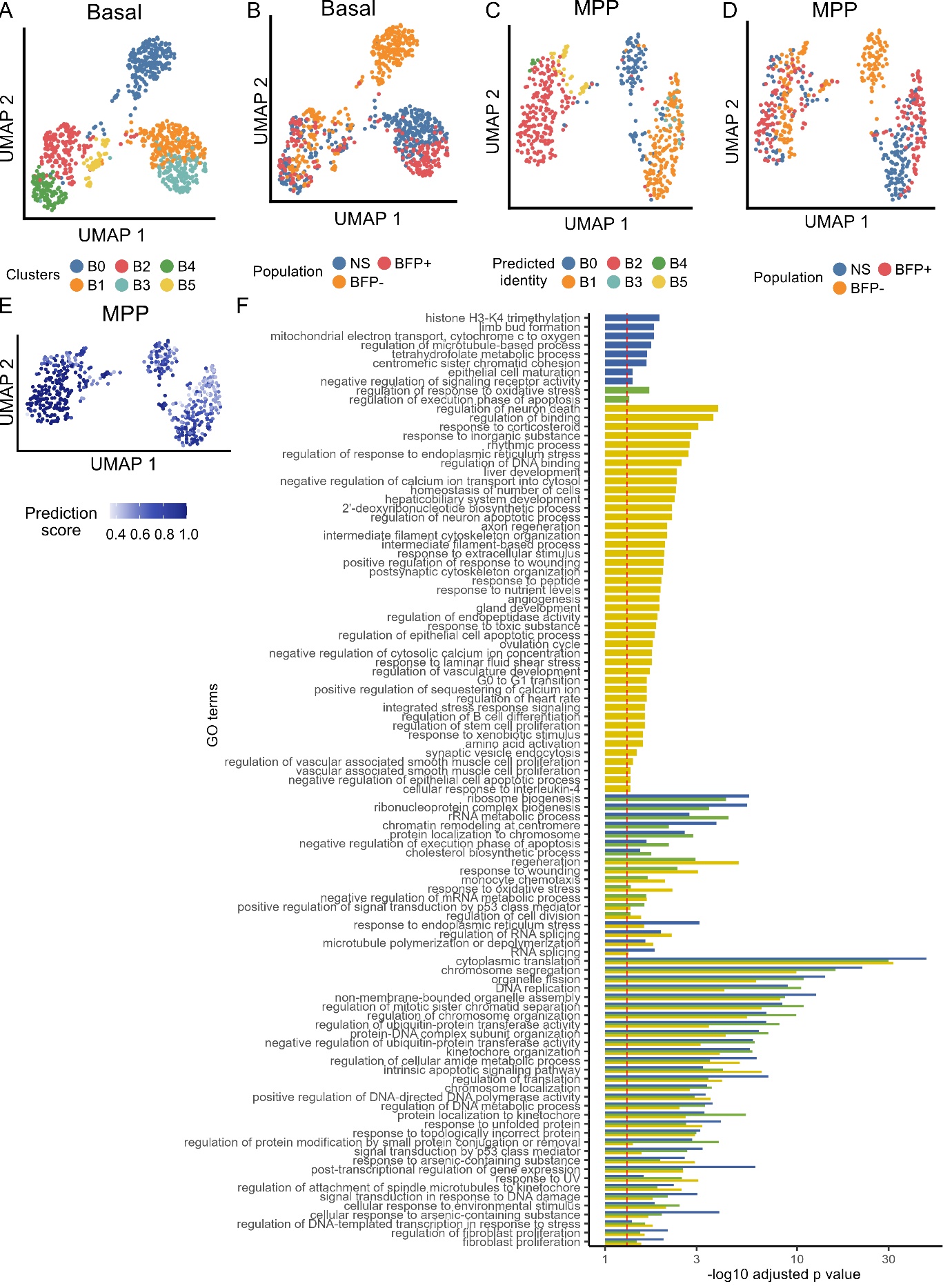


**Figure S4**. Effects of MPP^+^ to cells in different clusters (A) UMAP plot of basal control cells coloured by the cell clusters. Clusters are named according to size from B0 to B5 and annotated by cell type (3 progenitor and 3 neuronal, n = 871 cells). (B) UMAP plot of basal control cells coloured by sorting status (BFP^+^, BFP^-^ and NS). (C) UMAP plot of MPP^+^ treated cells with annotation predicted using the transcriptional profile of the basal clusters and coloured accordingly (n = 531). (D) UMAP plot of MPP^+^ treated cells coloured by sorting status. Stronger segregation is observed in neuronal clusters. (E) UMAP plot of MPP^+^ treated cells coloured by the prediction score of the re-annotation. (F) Simplified GO terms associated with upregulated genes in MPP^+^ treated neuronal clusters (B0, B1 and B3) of different sorting status.


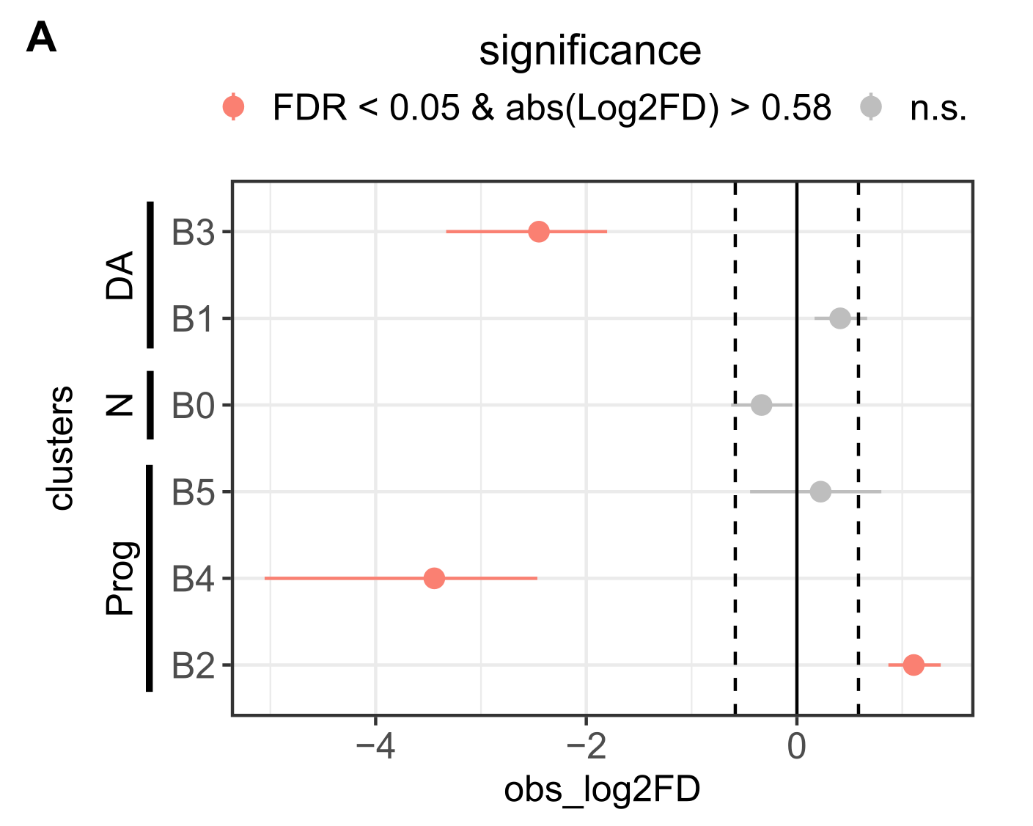


**Figure S5.** Proportion difference per cluster in basal and MPP^+^ conditions. The plot indicates the proportion difference in cell clusters between the basal and MPP^+^ conditions tested using a randomization test as implemented in scProportionTest. Confidence intervals are shown based on 10,000 randomizations. The absolute fold differences above 1.5 (dashed line) and the FDR < 0.05 are indicated in salmon colour while non-significant differences are shown in grey.

**Supplemental tables**

**Table S1. PCR primers.**

| *Primer ID* | *Forward 5’-3’* | *Reverse 5’-3’* | *Size(bp)* |
| --- | --- | --- | --- |
| AAVS1-5HR | CCTGGCCATTGTCACTTTGC | GGGCTATGAACTAATGACCCCG | 998 |
| AAVS1-3HR | ATTTTCATTGCAATAGTGTGTTGGA | TGGGGTCCAGGCCAAGTA | 990 |
| LMX1A-5HR | ACTTGAAGCAGAGGCAGAACATT | CAGGTTCTTGCGAACCTCATCAC | 943 |
| LMX1A-3HR | GGGAGGATTGGGAAGACAATAGC | CAGAATGAACCCCAGCACTCAA | 737 |
| LMX1A-WT | AGTTTCACCTGGAGCCTGTG | TGGGAAGTTGTTAGGAGTCCC | 590 |
| AAVS1-WT | CAAAGTACCCCGTCTCCCTG | ATCCTCTCTGGCTCCATCGT | 558 |

**Table S2. CNV analysis of the parental and engineered iPSC lines.**

| *Cell line* | *Genotype* | *3p14.2* | *3p13* | *6p22.3* | *18q22.1* |
| --- | --- | --- | --- | --- | --- |
| KOLF2-C1 | parental | Duplication | Deletion | Deletion | Duplication |
| A17 | BFP^+/+^CRE^-/-^ | Duplication | Deletion | Deletion | Duplication |
| A17L25 | BFP^+/+^CRE^+/-^ | Duplication | Deletion | Deletion | Duplication |
| A17L35 | BFP^+/+^CRE^+/-^ | Duplication | Deletion | Deletion | Duplication |
| A17L43 | BFP^+/+^CRE^+/+^ | Duplication | Deletion | Deletion | Duplication |
| A17L44 | BFP^+/+^CRE^+/+^ | Duplication | Deletion | Deletion | Duplication |

**Table S3. Primary antibodies used.**

| *Marker* | *Catalogue number* | *Dilution* |
| --- | --- | --- |
| BFP (tRFP) | AB233 Cambridge Biosciences | 1:3000 |
| NES | 611659 BD Biosciences | 1:300 |
| LMX1A | AB10533 Millipore | 1:2000 |
| FOXA2 | AF2400 R&D | 1:1000 |
| OTX2 | AF1979 R&D | 1:300 |
| EN1 | 4G11 DHSB | 1:100 |
| LMX1B | Custom made | 1:1000 |
| PAX6 | ab78545 Abcam | 1:1000 |
| TH | MAB318 Millipore | 1:500 |
| TH | AB152 Millipore | 1:500 |
| MAP2 | M4403 Sigma | 1:500 |
| Calbindin | CB38A Swant | 1:500 |
| GIRK2 | ab65096 Abcam | 1:200 |
| PITX3 | 38-2850 Invitrogen | 1:250 |
